# Small-angle scattering for beginners

**DOI:** 10.1107/S1600576721010293

**Published:** 2021-11-25

**Authors:** Cedric J. Gommes, Sebastian Jaksch, Henrich Frielinghaus

**Affiliations:** a Jülich Center for Neutron Science, Forschungszentrum Jülich GmbH, 52425 Jülich, Germany; b Jülich Center for Neutron Science at the Heinz Maier Leibnitz Zentrum, Forschungszentrum Jülich GmbH, Lichtenbergstrasse 1, 85747 Garching, Germany

**Keywords:** small-angle scattering, SAXS, SANS, form factors, structure factors

## Abstract

Many experimental methods are available for the characterization of nanostructures, but most of them are limited by stringent experimental conditions. When it comes to analysing nanostructures in the bulk or in their natural environment – even as ordinary as water at room temperature – small-angle scattering (SAS) of X-rays or neutrons is often the only option. The rapid worldwide development of synchrotron and neutron facilities over recent decades has opened unprecedented possibilities for using SAS *in situ* and in a time-resolved way. But, in spite of its huge potential in the field of nanomaterials in general, SAS is covered far less than other characterization methods in non-specialized curricula. Presented here is a rigorous discussion of small-angle scattering, at a technical level comparable to the classical undergraduate coverage of X-ray diffraction by crystals and which contains diffraction as a particular case.

## Abstract 2

A non-technical yet rigorous introduction to small-angle scattering is proposed, through the systematic use of Fresnel–Feynman analysis of interference phenomena.

## 1. Introduction

A major difficulty when working with nanostructures – synthesizing them, manipulating them, studying their properties *etc.* – is that we cannot see them with the naked eye. Or when we can, *e.g.* using electron microscopy, the observation conditions are very different from the sample’s natural conditions. Frozen, desiccated or metallized nano­structures in an electron beam may have little in common with their natural state swollen in water, undergoing thermal motion and surrounded by all types of ions. Moreover, the amount of material characterized in any microscopy observation is so small that one can always doubt whether it is representative of the sample as a whole. In many situations, therefore, workers in the field of nanomaterials have to rely on indirect characterization methods, whereby a macroscopic amount of sample is analysed in its normal environment but only incomplete structural information is obtained. Small-angle scattering (SAS) is such a method.

**Figure 1:**
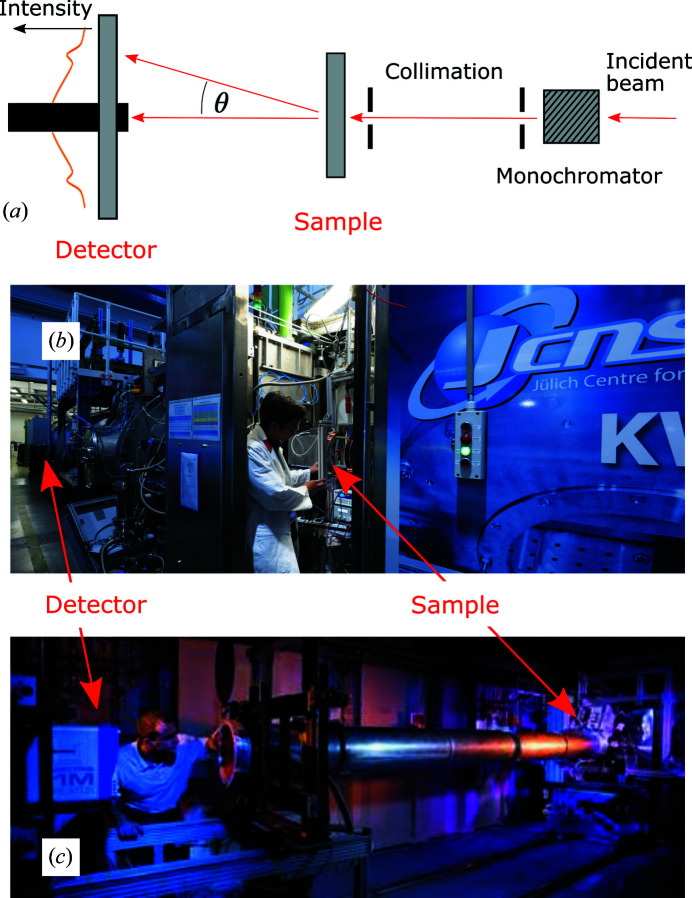
(*a*) A sketch of the main elements in a typical small-angle scattering instrument. (*b*) The KWS-1 small-angle neutron scattering instrument at the Heinz Meier-Leibnitz Zentrum (Feoktystov *et al.*, 2015 ▸). (*c*) The synchrotron small-angle scattering setup on the Dutch–Belgian beamline (BM26) of the European Synchrotron Radiation Facility (image courtesy of Professor B. Goderis). In both cases, the path of the beam is from the right to the left.

In its principle, SAS is very similar to better-known diffraction experiments (Guinier & Fournet, 1955 ▸; Glatter & Kratky, 1982 ▸). In its most popular form, an X-ray (small-angle X-ray scattering, SAXS) or neutron (small-angle neutron scattering, SANS) beam is shone on a sample and the intensity of the scattered beam is measured downstream as a function of the angle [Fig. 1 ▸(*a*)]. Accurate measurement of scattering at angles lower than a few degrees poses specific experimental difficulties (Schmatz *et al.*, 1974 ▸). In the present discussion, however, we focus on the principle of the method and on the structural significance of the measured signal. From that perspective, the main difference from diffraction experiments is that the angles investigated with SAS are much shallower. To some extent, SAS can be thought of as the analysis of the fine structure of what might be considered as the transmitted beam in a typical diffraction experiment. In order to resolve small scattering angles experimentally, the detector must generally be positioned far away from the sample, so SAS instruments are generally large [Figs. 1 ▸(*b*) and 1 ▸(*c*)].

**Figure 2:**
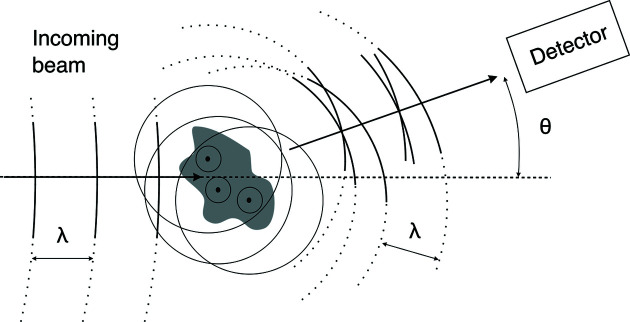
A Huygens-like description of a scattering experiment. When an incoming beam of wavelength λ is shone on a sample, each material point hit by it becomes the source of an isotropic secondary wave of identical wavelength. The intensity measured on a detector at any given angle θ results from the interference of all the secondary waves.

The physical principles underlying the SAS signal are sketched in Fig. 2 ▸. In the case of X-rays, any electron met by the incoming beam is accelerated by its oscillating electric field, and this results in the emission of secondary electromagnetic waves with identical frequency. In the case of SANS, the incoming neutrons interact with nuclei present in the sample via the strong nuclear force, and the scattering is a quantum-mechanical effect concerning the wavefunctions of both neutron and atomic nuclei. In the context of the Born approximation (Squires, 2012 ▸; Loh, 2017 ▸), however, each nucleus met by the neutron beam can also be thought of as the source of a secondary wavefunction. Therefore, although the physics of X-ray and neutron scattering are quite different, their geometric aspects can be discussed in terms of the same classical Huygens-like description of wave propagation in Fig. 2 ▸. In both cases, the intensity measured on the detector results from the interference of all secondary waves as a function of the scattering angle θ. Analysing SAS patterns consists of inferring structural information from the thus-measured intensities.

**Figure 3:**
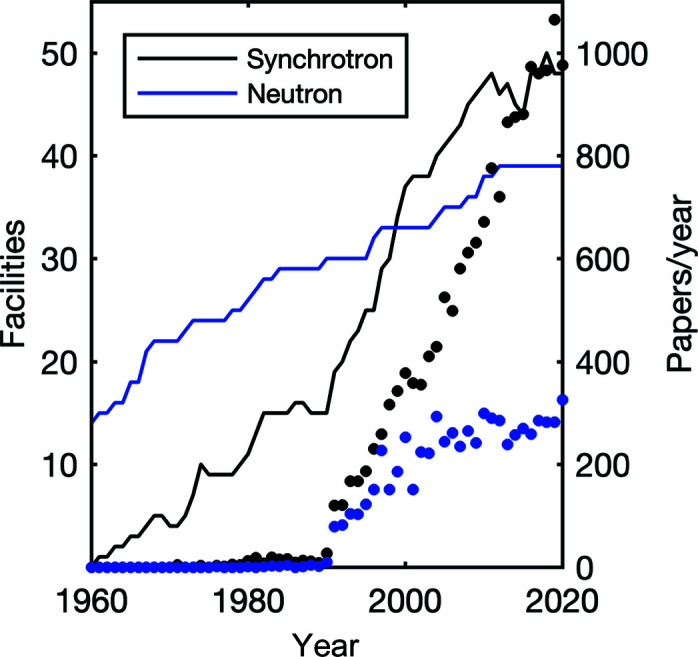
The number of synchrotrons and neutron facilities active worldwide, compiled from Wikipedia (https://en.wikipedia.org/wiki/List_of_synchrotron_radiation_facilities), Lightsources.org (https://lightsources.org/) and Neutronsources.org (https://neutronsources.org/neutron-centres/), and the number of papers published yearly on SANS (blue dots) or SAXS (black dots), compiled from the Web of Knowledge database (https://webofknowledge.com).

Although SAXS can be measured on commercial laboratory instruments, users of the technique also benefit from the worldwide development of large-scale facilities such as synchrotrons and free-electron lasers, many of which have instruments dedicated to small-angle scattering. Fig. 3 ▸ testifies to the booming of the field over the past few decades. Historically, starting from the 1960s, first-generation synchrotrons were particle accelerators built for physics research. As a consequence of the acceleration of charged particles, these instruments inevitably produced X-rays, but this was largely considered a nuisance. Second-generation synchrotrons were particle accelerators deliberately built as intense X-ray sources for characterization studies. From approximately 1990 to 2010, the worldwide development of optimized third-generation synchrotrons took place at the impressive pace of one new facility commissioned yearly, and this is continuing with the current development of free-electron lasers as a fourth-generation synchrotron source (Margaritondo & Rebernik Ribic, 2011 ▸). Since the 1960s, the brilliance of synchrotron sources has tripled every eighteen months (Rubensson, 2016 ▸), so that current synchrotron beams are billions of times more intense than X-rays emitted by, say, rotating anodes. In addition to higher intensity, synchrotron beams are also more coherent, better focused, with a broader energy spectrum available *etc.*, which enables a wealth of scattering experiments that are impossible on laboratory sources. The difference between synchrotron and laboratory X-rays is often likened to the difference between a laser and a light bulb.

Comparable progress has taken place in the field of neutron facilities, which developed at a fast pace all through the second half of the 20th century, based on both nuclear reactors and spallation sources (Rush, 2015 ▸). A major event in the field is the current development of the European Spallation Source, anticipated to become the most brilliant neutron source worldwide (Santoro *et al.*, 2020 ▸; Andersen *et al.*, 2020 ▸). The increasing number of facilities and brilliance of the sources has been accompanied by qualitative improvements in the instrumentation, with better optics, detectors, instrument control, data analysis tools *etc.* (Koch, 2010 ▸; Andersen *et al.*, 2020 ▸)

The fields of application of SAS are extremely numerous as it can in principle be applied to study any nanoscale system. The following are just a few examples, which are inevitably biased towards the authors’ scientific interests. Among others, SAS is routinely used to analyse nanostructures in solution, which is invaluable for colloids and proteins (Tuukkanen *et al.*, 2017 ▸; Gräwert & Svergun, 2020 ▸). Nanostructures can also be analysed in working conditions, while materials are being mechanically deformed (Pawlak & Galeski, 2005 ▸) or submitted to a variety of thermal, chemical or electromagnetic stimuli (Bailey, 2003 ▸; Hamley *et al.*, 2004 ▸; Fujii *et al.*, 2012 ▸). Nanostructures can also be studied under extreme experimental conditions. Spectacular examples are the *in situ* analysis of nanostructured soot formation in flames (di Stasio *et al.*, 2006 ▸) and fireball lightning (Mitchell *et al.*, 2008 ▸). SAS also enables biochemical structures and processes to be studied in biological tissues *in vivo* at the nanometre scale, such as the spinning of spider silk (Riekel & Vollrath, 2001 ▸) or muscle contraction (Ait-Mou *et al.*, 2016 ▸).

The availability of different types of beams for performing SAS experiments adds to the versatility of the method. In addition to X-rays and neutrons, scattering experiments can also be done with electrons, visible light or muons (obtained as by-products of neutrons in spallation sources) (Windsor, 1988 ▸; Pynn, 1990 ▸). The various probes differ in the sample characteristics they are sensitive to, yielding different types of contrasts. In the case of neutrons, this capability is further expanded through the possibility of isotope substitution. Another important characteristic of a probe is its penetration depth, which may enable or forbid certain types of experiment. The different brilliance of the sources is also central for choosing a probe for time-resolved studies *etc.* Often, different types of scattering technique are combined, each of which provides different and complementary information on the investigated system. (Allen *et al.*, 2007 ▸; Whitten & Trewhella, 2009 ▸; Genix & Oberdisse, 2015 ▸).

Despite its huge potential as a characterization technique for nanostructures in general – across the fields of chemistry, physics, biology and materials science – SAS remains relatively unpopular in most scientific curricula compared with other experimental methods. This is partly due to its reputation as a very indirect technique, the thorough understanding of which requires a strong taste for mathematics. The aim of the present paper is to show that most SAS data can be analysed qualitatively, yet rigorously, with minimal mathematical background. The reader is referred to excellent monographs (Guinier & Fournet, 1955 ▸; Glatter & Kratky, 1982 ▸; Feigin & Svergun, 1987 ▸), textbooks (Sivia, 2011 ▸) and other educational material (Schmatz *et al.*, 1974 ▸; Zaccai & Jacrot, 1983 ▸; Windsor, 1988 ▸; Pynn, 1990 ▸; Hammouda, 1995 ▸; Roe, 2000 ▸; Jaksch, 2019 ▸; Hamley, 2021 ▸) for a more thorough, but also more technical, coverage of the subject.

## 2. Structural significance of small-angle scattering

For reasons that will soon be clear, SAS intensities are plotted not against the scattering angle θ but against the magnitude of the scattering wavevector *q*, defined as 

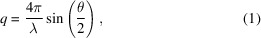

where λ is the wavelength of the X-rays or neutrons. The physical meaning of *q* is that ℏ*q* is the momentum transfer to the photon or neutron during the scattering event. In the context of diffraction studies, the scattering angle is sometimes defined as 2θ instead of θ. In that context, *q* is therefore defined as being proportional to sin(θ), but this is the same physical quantity. Among other advantages, scattering patterns plotted against *q* are independent of the particular wavelength selected for the experiment, which would not be the case if they were plotted against θ.

**Figure 4:**
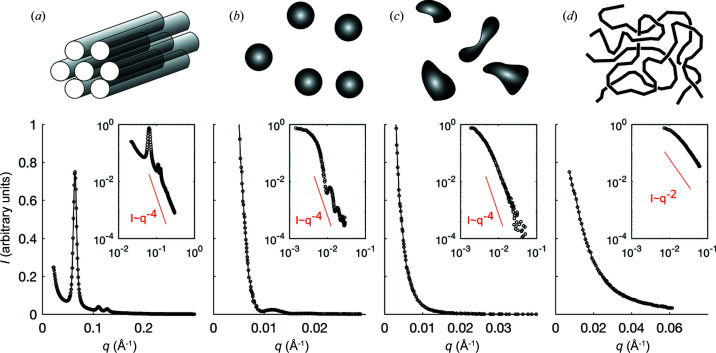
Representative SAS patterns. (*a*) SAXS of ordered mesoporous silica (Gommes *et al.*, 2016 ▸), (*b*) SANS of a colloidal suspension of latex nanoparticles (Hammouda, 1995 ▸), (*c*) SAXS of a laboratory model of fireball lightning (Mitchell *et al.*, 2008 ▸), and (*d*) SANS of a blend of deuterated and protonated polystyrene (Hammouda, 1995 ▸). The insets show the same data on double logarithmic scales and compare them with power laws of the type *I* ≃ *q* ^−4^ and *I* ≃ *q* ^−2^. Qualitative sketches of the structures are provided at the top.

Fig. 4 ▸ displays four data sets that are representative of qualitatively different types of scattering pattern that one often encounters when applying SAS. For now, we only highlight their most distinctive features, which we will discuss and explain in detail in the rest of the paper. Fig. 4 ▸(*a*) is a SAXS pattern measured on ordered nanoporous silica, consisting of a hexagonal array of cylindrical pores a few nanometres across (Gommes *et al.*, 2016 ▸). The scattering peaks can be interpreted in terms of diffraction by the periodic pore structure, but with a periodicity in the nanometre range corresponding to the distance between the pores. Many materials do not exhibit periodicity on the nanometre scale and their SAS patterns do not exhibit any sharp peak. One such pattern is plotted in Fig. 4 ▸(*b*), which was measured by SANS on an aqueous colloidal suspension of latex nano­particles (Hammouda, 1995 ▸). The pattern seems featureless on linear scales, except for a slight hump around *q* = 0.01 Å^−1^. On logarithmic scales, however, the scattered intensity exhibits a plateau at small *q*, followed by an oscillatory decrease. Fig. 4 ▸(*c*) displays a SAXS pattern obtained by shining a synchrotron beam through a laboratory model of fireball lightning (Mitchell *et al.*, 2008 ▸). The SAXS pattern is dominated by an *I* ≃ *q* ^−4^ trend at large values of *q*, which appears as linear on the double logarithmic scales of the inset. The last pattern [Fig. 4 ▸(*d*)] was obtained by neutron scattering on a 50:50% blend of deuterated and protonated polystyrene, which provides strong scattering contrast to half of the polymer chains (Hammouda, 1995 ▸). This pattern too exhibits an overall power-law scattering that levels off towards smaller values of *q*, but the scattering exponent is here close to 2.

In the context of scattering, it is difficult for anybody with a scientific education not to think of Bragg’s law, and this can be misleading in the context of SAS. Bragg’s law is concerned with diffraction, which is a very specific scattering phenomenon that happens only with periodic structures. In that case, the beam is scattered exclusively at well defined diffraction angles θ_*d*_, which are related to the periodicities *d* of the structure via (Sivia, 2011 ▸; Loh, 2017 ▸) 

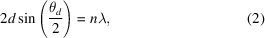

where *n* is any positive integer. Before discussing SAS, it is important to emphasize that Bragg’s law is not a general law of scattering but a consequence of the general principles of Fig. 2 ▸, when particularized to spatially periodic structures. In the specific case of Fig. 4 ▸(*a*), one can use equation (2) to infer the spacing between pores from the positions of the peaks, but we shall see that the scattering pattern contains much more structural information than that. Moreover, equation (2) is irrelevant and useless in cases of non-periodic structures, such as in Figs. 4 ▸(*b*)–4 ▸(*d*).

Analysing from Fig. 2 ▸ the general conditions for destructive or constructive interference of all secondary waves when they reach the detector, in relation to all possible sizes and shapes of the electron- or nucleus-containing nanostructures, may seem at first to be a difficult task. The following two observations make it simpler. First, under the conditions of SAS the emission of secondary waves can be assumed to be isotropic (Glatter & Kratky, 1982 ▸; Sivia, 2011 ▸). Second, provided the sample is sufficiently thin the secondary waves reach the detector without being scattered a second time (Frielinghaus, 2018 ▸), so it is only the unperturbed incoming beam that is responsible for the secondary waves. As a consequence, for any given scattering angle θ, that is for a given pixel of the detector, the phase ϕ of a specific secondary wave reaching the detector depends *only* on the position (*x*, *y*, *z*) of the scattering centre. In other words, for any given angle θ one can calculate a phase map ϕ_θ_(*x*, *y*, *z*) which is a characteristic of the instrument. That function is the phase of the secondary wave that would reach the detector in the event that the sample had a scattering centre at a point (*x*, *y*, *z*). The scattered intensity is then obtained as a second step, by comparing the sample-independent phase map ϕ_θ_(*x*, *y*, *z*) with the actual spatial distribution of electrons or nuclei in the considered sample. The phase map can be thought of as the spectacles through which any sample is analysed in a SAS experiment. It is therefore occasionally referred to as the probe wave (Windsor, 1988 ▸). The phase map is different for each scattering angle, which is why a full scattering pattern measured over a wide angular range provides rich structural information.

**Figure 5:**
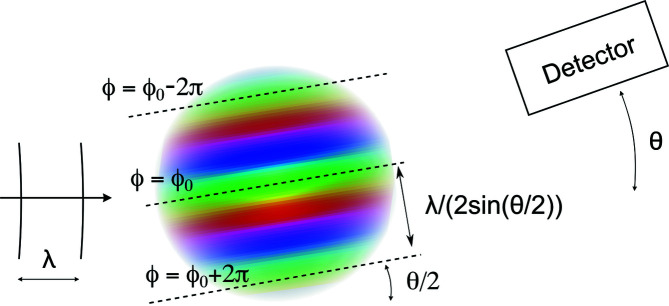
For a given scattering angle θ, the phase map ϕ_θ_(*x*, *y*, *z*) is the phase of the secondary wave that would reach the detector in the event that the sample had a scattering centre (electron or nucleus) at a point (*x*, *y*, *z*). The phase map takes constant values on planes at an angle θ/2 compared with the incoming beam, and the distance between planes with identical phases (modulo 2π) is 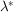 = λ/[2sin(θ/2)].

Calculating the phase map for a given angle ϕ_θ_(*x*, *y*, *z*) is a purely geometric question. Starting from any given point (*x*, *y*, *z*) two half lines are drawn, one towards the source and the other towards the detector. Their lengths are the distances travelled by the primary and secondary waves, respectively. Let us call the sum of the two lengths *L*(*x*, *y*, *z*). Because the scattering is instantaneous and the phase of a wave increases by a quantity 2π each time the wave travels a distance equal to its wavelength λ, the phase map is obtained by multiplying *L*(*x*, *y*, *z*) by 2π/λ. The calculation of *L*(*x*, *y*, *z*) is explained in Appendix *A* [App appa] on the basis of simple trigonometry. The result is sketched in Fig. 5 ▸: the phase map ϕ_θ_(*x*, *y*, *z*) is found to take constant values on geometric planes oriented at an angle θ/2 with respect to the incident beam. As a consequence, the phase depends only on the space coordinate perpendicular to the planes, which we call *y*. With that convention, the result is written as 

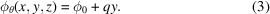

Here ϕ_0_ is an irrelevant constant that depends on the arbitrarily chosen origin of *y*, and *q* is the same as in equation (1). Equation (3) shows that ϕ_θ_(*x*, *y*, *z*) increases by 2π over a distance 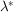 given by 

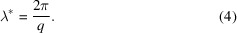

The notation 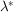 highlights the fact that this length plays the role of an apparent, and θ-dependent, wavelength. For SAS one can approximate sin(θ/2) ≃ θ/2 in the definition of *q*, so that 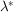 is approximately inversely proportional to the scattering angle, 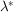 ≃ λ/θ, with θ expressed in radians. In other words, the smaller the angle, the larger the size of the investigated objects. For X-rays and thermal neutrons with λ in the ångström range, a typical angle of 1° ≃ 0.017 rad converts to 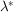 ≃ 6 nm. This is the reason why small-angle scattering is a suitable experimental technique to probe nanometre-scaled structures.

The concept of a phase map shown in Fig. 5 ▸, together with its quantitative relation to the observation angle θ in equation (4), is all that is needed for a qualitative understanding of SAS. Before proceeding, however, another caveat is necessary. Although Fig. 5 ▸ superficially resembles the classical textbook discussions of Bragg’s law – where the crystal lattice planes are indeed parallel to each other and oriented at an angle of θ/2 with respect to the incoming beam – the planes we are dealing with here are by no means material planes. They are the geometric locus of points that would lead to secondary waves reaching the detector in phase, in the event that the considered sample had scattering centres (electrons or nuclei) there.

## 3. Scattering by individual particles: the form factor

We consider a single nanoparticle irradiated by X-rays and a detector at some angle θ, but the discussion also holds for neutrons, provided electrons are replaced by nuclei. The particle is assumed to be homogeneous so that the number of electrons per unit volume is the same everywhere in it. If the particle is made of silica, for example, every cubic nanometre of it contains about 700 electrons. Each of these electrons is the source of a secondary wave, which is described as a complex number *a*exp(*i*ϕ) when it reaches the detector. The amplitude *a* is the same for all electrons, but the phase ϕ depends on the electron position through ϕ_θ_(*x*, *y*, *z*). The amplitude *A* of the resulting wave is the sum of the contributions of all electrons in the particle, namely 

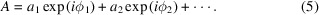

This sum has a simple geometric interpretation in the complex plane, whereby each term is associated with an arrow of length *a* and angle ϕ, which are then added head-to-tail. This type of analysis goes back to the early introduction of complex numbers into optics by Fresnel (Karam, 2018 ▸) and was popularized notably by Feynman (1985 ▸) in his lectures on quantum electrodynamics.

**Figure 6:**
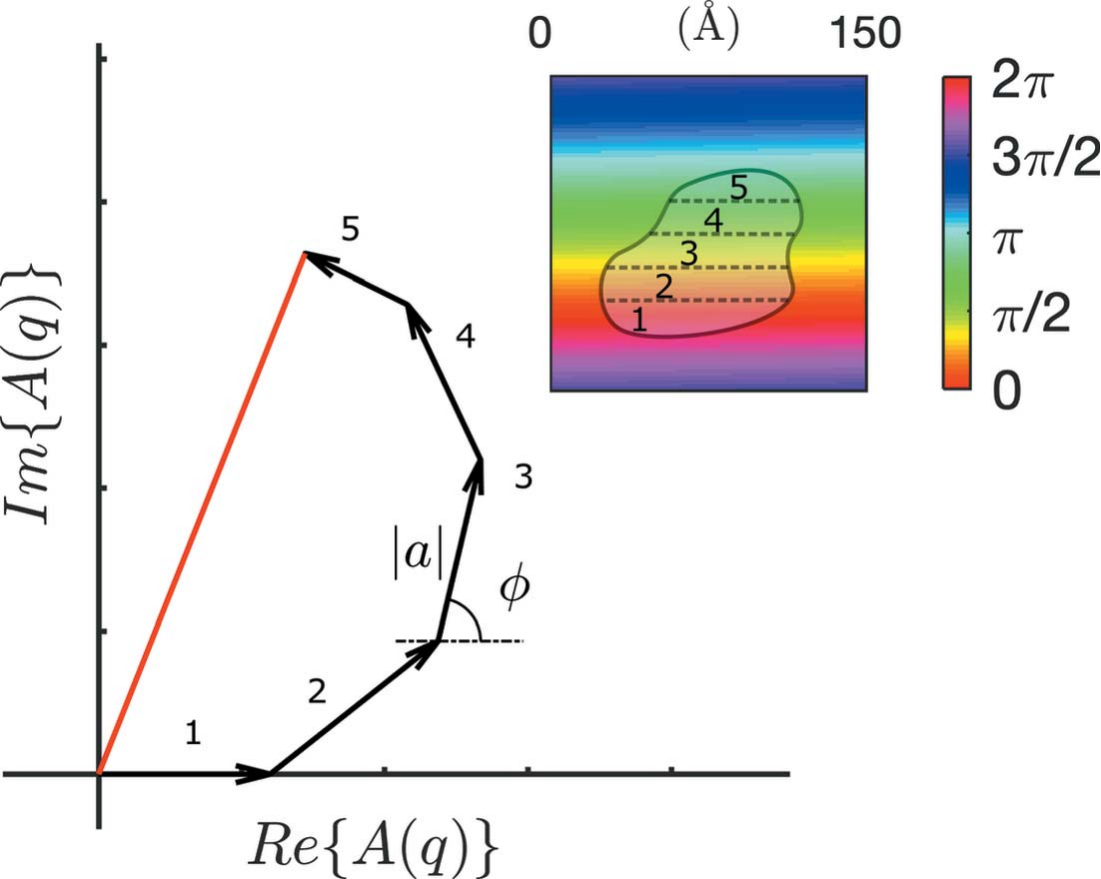
Phase-map analysis of the amplitude *A*(*q*) of the wave scattered by a nanoparticle. The inset displays the nanoparticle overlaid with ϕ_θ_(*x*, *y*, *z*) for the particular value *q* = 0.042 Å^−1^, corresponding to 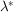 ≃ 150 Å. Each slice of the particle (numbered 1 to 5) is assigned a complex number with phase ϕ and modulus |*a*| proportional to the slice volume. The amplitude of the scattered wave (in red) is obtained by adding the contributions of all slices, as in equation (5).

Equation (5) may apply to all electrons individually, in which case all *a* values are equal, but it is more convenient to calculate the sum by grouping the contributions of electrons with identical phases, which are all located within slices oriented at an angle θ/2 with respect to the incoming beam (see Fig. 5 ▸). With such a procedure, and assuming that equal volumes of material contain equal numbers of electrons, equation (5) still applies with *a* proportional to the volume of the slice and ϕ equal to the constant value of ϕ_θ_ within the slice. The resulting interference is illustrated in Fig. 6 ▸ for an arbitrarily shaped particle cut into five slices.

For the analysis to be mathematically accurate, the slices have to be made infinitely thin, which leads to the following expression for the scattered intensity (Guinier & Fournet, 1955 ▸; Glatter & Kratky, 1982 ▸; Feigin & Svergun, 1987 ▸; Pynn, 1990 ▸; Sivia, 2011 ▸; Jaksch, 2019 ▸):

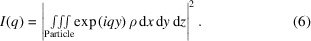

In this equation, the imaginary exponential accounts for the phase *qy* of the secondary waves originating in all electrons at a point (*x*, *y*, *z*) in line with equation (3), ρ is the electron density of the material that makes up the particle, so that ρ d*x* d*y* d*z* is the number of electrons in an infinitesimal volume, and the integral replaces the sum in equation (5) and is responsible for the interference. Finally, we note that detectors cannot measure the amplitude of a wave but only its intensity, defined as the squared modulus *I* = |*A*|^2^. Equation (6) is valid for neutron scattering as well, provided the electron density ρ is replaced by the neutron scattering-length density, which characterizes how strongly the nuclei that make up the particle interact with neutrons (Pynn, 1990 ▸).

Equation (6) is usually stated by saying that the scattered intensity is the squared modulus of the Fourier transform of a material’s electron density. Although this may sound mathematically advanced, many aspects of it can be understood qualitatively yet rigorously from the simpler perspective of equation (5), through the geometric interpretation of complex numbers in Fig. 6 ▸. For small values of *q*, the periodicity of the phase map 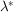 = 2π/*q* is much larger than the size of the particle, so that all scattering centres of the particle scatter in phase. In Fig. 6 ▸, this would correspond to a situation where all the arrows point in the same direction, which maximizes the scattered intensity. By contrast, for large values of *q* the length 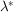 is smaller than the particle, so that different parts of the particle scatter out of phase. In Fig. 6 ▸ this corresponds to arrows pointing in different directions, which results in a lower scattered intensity. This simple analysis explains the overall shape of most scattering patterns in the insets of Fig. 4 ▸: they are globally decreasing functions with a plateau at small *q*, and the transition occurs where 2π/*q* is comparable to the size of the scattering objects.

**Figure 7:**
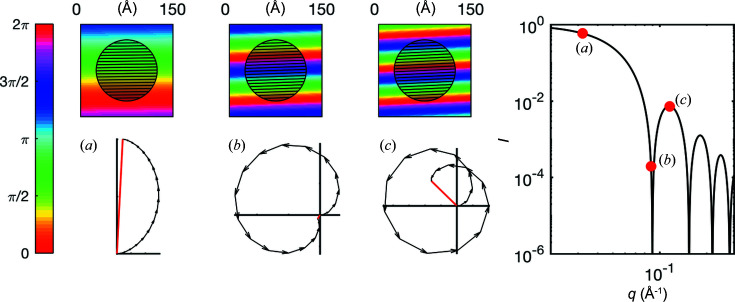
Small-angle scattering by a spherical nanoparticle of radius *R* = 50 Å. The phase maps ϕ_θ_(*x*, *y*, *z*) corresponding to *q* values of (*a*) 0.03 Å^−1^, (*b*) 0.09 Å^−1^ and (*c*) 0.12 Å^−1^ are represented in colour. For any value of *q*, the nanoparticle is mentally split into slices with uniform phases, and the resulting complex amplitudes are added (as in Fig. 6 ▸). The resulting scattering pattern, given by equation (8), is shown on the right.

The intensity *I*(*q*) scattered by an individual nanoparticle over a complete range of *q* is referred to as its form factor (Pynn, 1990 ▸; Sivia, 2011 ▸) and constitutes its SAS fingerprint. It is customary to factor out the effect of the electron density and that of the particle volume *V*, so that the form factor *P*(*q*) is defined as 

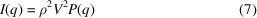

and satisfies *P*(*q*) = 1 for small *q*. From equation (6), the form factors can be calculated for a variety of particle shapes – spheres, ellipsoids, cylinders, platelets, spherical shells *etc.* – and mathematical expressions can be found in the literature (Pedersen, 1997 ▸). As it is often desirable to assign a single size parameter to investigated structures, one is naturally led to modelling particles as spheres. The form factor of a sphere of radius *R* is given by 

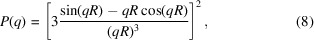

which is plotted in Fig. 7 ▸ for the particular value *R* = 50 Å. The form factor of a sphere exhibits a distinctive oscillatory decrease with alternating maxima and minima of *P*(*q*), as notably observed in the inset of Fig. 4 ▸(*b*). The first minimum in the form factor corresponds to the value of *q* where the numerator of equation (8) vanishes, and it is found to be related to the radius of the nanoparticles via *R* ≃ 4.5/*q*. In the case of Fig. 4 ▸(*b*), the experimental value *q* = 0.009 Å^−1^ converts to a particle radius of *R* = 50 nm, which is indeed the size of the latex nanoparticles in that sample.

The oscillatory shape of the form factor of a sphere can be understood through the same geometric construction as in Fig. 6 ▸. This is illustrated in Fig. 7 ▸ for a given sphere and three different values of *q*. In the figure, the sphere is cut into slices with uniform phases and the corresponding amplitudes are added to form a meandering curve in the complex plane. The case of Fig. 7 ▸(*a*) is relatively similar to Fig. 6 ▸. It is representative of low-*q* scattering, where all points of the sphere lead to secondary waves with similar phases. When *q* is progressively increased, destructive interference builds up, whereby different parts of the sphere lead to distinctly different phases. This corresponds to a curve that curls in the complex plane. For the specific value *q* ≃ 4.5/*R* the curling is such that the curve closes head-to-tail, which is the origin of the first minimum [Fig. 7 ▸(*b*)]. Increasing *q* further, the end of the curve moves away from its starting point, until a maximum is reached corresponding to Fig. 7 ▸(*c*) *etc*. A movie is provided as supporting information to illustrate this in an animated way.

If the nanoparticles in a sample do not all have the same exact size or shape, the conditions for constructive and destructive interference differ from one particle to the next. Therefore, the presence of sharp oscillations in an experimental SAS pattern testifies to a narrow size distribution. This situation contrasts with Figs. 4 ▸(*c*) or 4 ▸(*d*), which exhibit a scattered intensity that decreases continuously with *q*, as is typical of polydisperse systems. When plotted on double logarithmic scales (inset) the scattered intensities display two regimes: a plateau at low *q* followed by a decreasing intensity, with the transition happening for 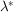 comparable to the particle size. From equation (6) it can be shown that, for a single particle, the progressive onset of destructive interference for small values of *q* obeys a universal law that is independent of the particle shape, namely (Guinier & Fournet, 1955 ▸; Glatter & Kratky, 1982 ▸; Feigin & Svergun, 1987 ▸; Sivia, 2011 ▸; Jaksch, 2019 ▸) 

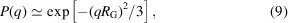

where *R*_G_ is the radius of gyration of the particle. The latter is defined such that 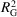 is the average squared distance between any point of the particle and its centre of mass. Equation (9) is known as Guinier’s law, and it provides a universal and model-independent way of determining particle sizes from SAS data. Plotting experimental scattering data as 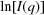 against *q* ^2^ often yields a linear trend at low *q*, the slope of which is 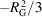 . Assuming specific types of structures, the *R*_G_ thus obtained can then be converted to more intuitive measures of the particle size. For example, the radius of a sphere is *R* = (5/3)^1/2^ *R*_G_; the length of a rod is *L* = 2(3^1/2^)*R*_G_; in a linear polymer chain with segments of length *b*, the number of segments is 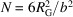 *etc.* (Glatter & Kratky, 1982 ▸; Sivia, 2011 ▸).

In addition to sizes, qualitative structural information is obtained by analysing the building up of destructive interference when *q* is increased beyond the limit of validity of equation (9), that is when the periodicity of the phase map 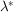 is made smaller than the particle size. This typically leads to power laws of the type *I* ≃ *q* ^−α^, which can easily be identified as straight lines with slope −α on double logarithmic plots. In the insets of Fig. 4 ▸ the scattering exponent is α = 4 for the first three samples and α = 2 for the last one. The specific exponent 4 is referred to as Porod’s law and is universal to all structures with clear-cut interfaces (Ciccariello *et al.*, 1988 ▸). Porod scattering was expected in the case of the nanoporous solid in Fig. 4 ▸(*a*), as well as that of the colloidal particles in Fig. 4 ▸(*b*). However, its experimental observation in the case of the fireballs [Fig. 4 ▸(*c*)] is proof that they contain compact nano­structures with well defined surfaces. By contrast, the exponent 2 observed in Fig. 4 ▸(*d*) points to a qualitatively different type of structure. Exponents close to 2 are often encountered for polymers, the structure of which on the nanometre scale consists of flexible strands folding and coiling randomly. When comparing loose and disordered structures as in Fig. 4 ▸(*d*) with dense and compact structures as in Fig. 4 ▸(*b*), it is intuitively understandable why the former should lead to a slower building up of destructive interference than the latter, when *q* is increased.

Discussing rigorously the relation between scattering exponents α and specific types of structure can only be done through the application of equation (6) to structural models. A broad array of such models are discussed in the SAS literature (Pedersen, 1997 ▸), and new models are regularly being developed each time a new type of material is encountered, with characteristics that are not captured by earlier models. Beyond the type of compact objects that universally lead to Porod scattering with exponent α = 4, other structures include infinitely thin needles and platelets, fractal-like hierarchical aggregates, random flexible polymers, persistent polymers, branched polymers *etc*. All of these lead to specific scattering exponents, as summarized in Table 1 ▸. Experienced users of SAS have these models in mind when they analyse experimental scattering patterns. When plotting the data on double logarithmic scales, as in the insets of Fig. 4 ▸, one can determine at a glance both the qualitative type of structure, via the exponent α, and its approximate size, via the cutoff value of *q* where destructive interference sets in. A scattering exponent does not point to a unique type of structure, as α = 2 might point either to a polymer coil or to randomly oriented platelets. In the context of a specific type of material, however, this is seldom ambiguous.

## 4. Scattering by collections of particles: the structure factor

The discussion has focused so far on the scattering by a single particle. This has overlooked the fact that any sample contains a large number of particles, each of which contributes to the scattered intensity, with possibly constructive or destructive inter-particle interference. The contribution of a collection of particles to the scattering is captured by the structure factor, which can also be analysed through equation (5). For that purpose, the sum of the secondary waves from all electrons or nuclei in the system is decomposed into the contributions of all individual particles. The contribution of each particle is a complex number *a*exp(*i*ϕ), with an amplitude given by the form factor through |*a*| = ρ*V*[*P*(*q*)]^1/2^ and a phase ϕ that depends on the position of the particle. If all particles are identical the amplitude can be factored out from the sum, which leads to the following expression for the scattered intensity: 

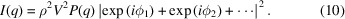

In this equation, the phase ϕ_*i*_ of the *i*th particle is the value of the phase map ϕ_θ_(*x*, *y*, *z*) at the position of, say, its centre of mass. When comparing with equation (7), the presence of many particles and their spatial distribution are found to be accounted for by an additional factor, which is generally *q* dependent, through the angular dependence of the phase map.

**Figure 8:**
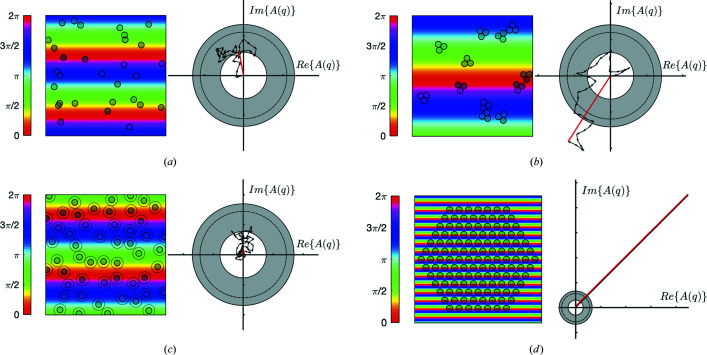
Interference patterns resulting from multiple-particle scattering, for the cases of (*a*) randomly positioned particles, (*b*) aggregated particles, (*c*) particles repelling each other and (*d*) a hexagonal crystalline structure. In each case, a typical phase map is shown with the particles in grey. In the complex-plane representation of wave amplitude, the contribution of each particle is shown as a black arrow and the resulting wave amplitude is in red. The grey annular area is the 50% confidence interval for the wave amplitude in the case where each particle has a random phase (incoherent scattering), and the dashed circle is the root-mean-square value.

Consider first the case where particles are randomly positioned in space [Fig. 8 ▸(*a*)], which is a fair approximation for dilute suspensions. In that case, the random position of each particle converts to a random orientation of its vector contribution to the wave amplitude, independent of the considered *q*. In other words, the amplitude of the scattered wave results from a random walk in the complex plane, whereby *N* successive steps are made with the same length *a* and random directions. As a consequence of the random orientation of each step, the average length of the walk is not proportional to the number of steps. Instead, the average squared length is proportional to *N* [see *e.g.* ch. 41 of Feynman *et al.* (2010 ▸)]. In the present situation, this means that the averaged squared amplitude of the wave, *i.e.* the scattered intensity, is equal to 

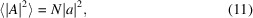

where |*a*|^2^ is the intensity scattered by a single particle, namely ρ^2^ *V* ^2^ *P*(*q*), and *N* is the number of particles. The scattered intensity per unit volume of the sample irradiated by X-rays can then be written as 

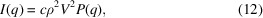

where *c* is the particle concentration in the suspension. Such proportionality of the intensity with the number of scatterers (or with their concentration) is typical of *incoherent* scattering, whereby all scatterers have uncorrelated phases. Note the difference from the form factor in Fig. 7 ▸ and equation (7), for which the intensity is proportional to (ρ*V*)^2^, that is to the squared number of electrons in each particle. A central characteristic of equation (12) is that the scattered intensity is proportional to the form factor, which is typical of dilute systems.

**Figure 9:**
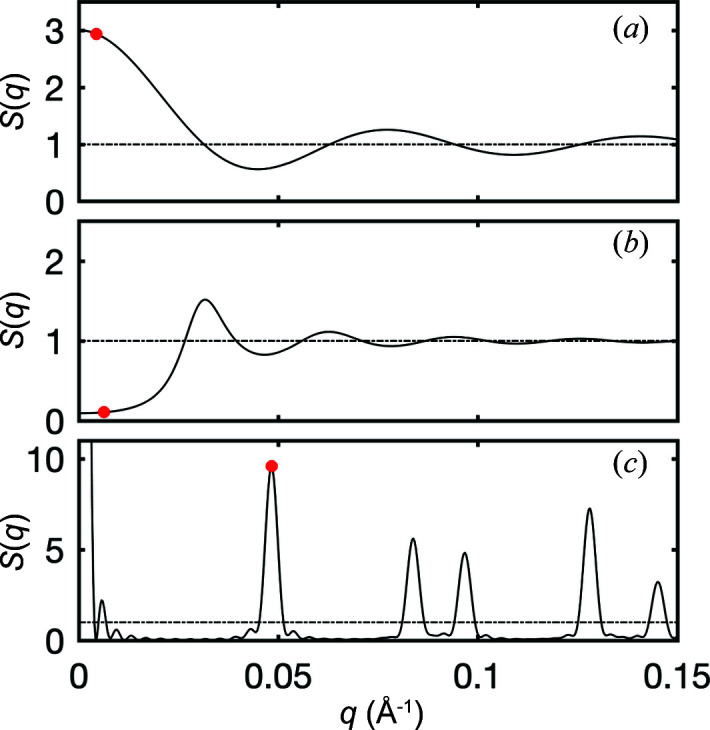
Structure factors corresponding to the structures in Fig. 8 ▸ with a particle radius of *R* = 50 Å. The structures are (*a*) aggregates of three touching particles, (*b*) particles repelling each other through hard-sphere interaction with a hard-sphere radius of 2*R* and (*c*) a 2D hexagonal periodic arrangement of particles with a lattice parameter of 3*R*. The dashed lines are at *S* = 1, corresponding to incoherent scattering, and the red dots indicate the specific values of *q* shown in Fig. 8 ▸.

In most situations the particle positions are not independent of one another, and the random-walk analysis of Fig. 8 ▸(*a*) [leading to equation (12)] has to be adapted. Fig. 8 ▸(*b*) illustrates the case of particle aggregation, for aggregates with *n*_*a*_ = 3 particles. The particular value of *q* chosen for the figure is such that 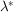 = 2π/*q* is larger than the aggregates, so that the phases ϕ of all particles within a given aggregate are statistically similar. This results in a correlated random walk in amplitude space, whereby each step in the walk is likely to have an orientation similar to the others. This leads to more elongated trajectories and to a larger intensity of the scattered wave. This can easily be understood by considering the limit of vanishingly small *q*, for which 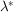 is much larger than the aggregates. In that limit all particles within an aggregate scatter coherently, so the length of a step is no longer *a* but *n*_*a*_ *a*. On the other hand, aggregates themselves scatter incoherently, so it is still a random walk but with only *N*/*n*_*a*_ steps. The modified version of equation (11) is then 

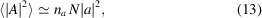

which shows that particle aggregation results in an *n*_*a*_-fold increase in the scattered intensity in the limit of vanishingly small *q*. For finite *q* the multiplying factor depends on the shape and size of the aggregates. The modified version of equation (12) is then 

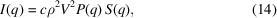

where *S*(*q*) is referred to as the structure factor. The specific structure factor obtained by evaluating the sum in equation (10) for the considered type of aggregates is plotted in Fig. 9 ▸(*a*). It does indeed satisfy *S* = 3 for small *q* and converges to *S* = 1 for large *q*, and the transition occurs when 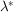 is comparable to the size of the aggregates. When 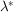 is much smaller than the aggregates, the phases of the contributing particles become uncorrelated and one recovers the situation described in equation (11). In addition to particle aggregation, SAS researchers have considered a wide variety of qualitatively different spatial statistics, and many analytical expressions are available for the corresponding structure factors (Pedersen, 1997 ▸).

Another common situation is the case where particles repel each other, which is relevant to concentrated suspensions. In the case of Fig. 8 ▸(*c*) this is modelled as a hard-sphere inter­action, whereby the particles cannot approach each other closer than twice their radii [see *e.g.* Kinning & Thomas (1984 ▸)]. The repulsion in real space converts to particles being unlikely to have similar phases. In that case, the random-walk analysis of the scattered amplitude still holds but the directions of the steps are anti-correlated. This results in a scattered wave amplitude lower than in the incoherent case, which is referred to as a correlation-hole effect. The structure factor corresponding to this situation is plotted in Fig. 9 ▸(*b*). In the limit of low *q*, similar to the case of Fig. 8 ▸ the structure factor is much smaller than one. In the limit of large *q*, corresponding to small 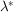 , minute differences in the particle positions lead to huge differences in their phases. The random-walk analysis becomes valid again in that limit, and *S*(*q*) converges to one.

The last case we consider is that of a periodic arrangement of particles, as in Fig. 8 ▸(*d*). The figure corresponds to a very specific value of *q* and a specific orientation, for which the periodicity of the structure coincides with direction *y* of the phase map. In this exceptional configuration, all particles in the structure scatter coherently. This is manifest in Fig. 8 ▸(*d*), where all the vectors are parallel and lead to very strong scattering. This corresponds to a diffraction condition, which is obtained when the periodicity of the phase map 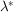 coincides with the spacing *d* between planes in a crystalline arrangement. Using then equation (1) to express this in terms of the scattering angle, one recovers Bragg’s law in the familiar form of equation (2). The integer *n* results from the observation that *n* 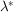 is also a periodicity of the phase map for any *n*. Scattering by a crystalline structure is not limited to the diffraction peaks. However, the intensity of the peaks scales like the squared number of particles, as for any coherent effect. On the other hand, the scattering between the peaks is incoherent. It therefore scales linearly with the number of particles and is hence much weaker than the peaks. Actual crystals have finite sizes and contain structural defects, both of which contribute to widening the scattering peaks beyond Bragg’s condition. In the limit of large *q*, all the thus-broadened peaks overlap and *S*(*q*) converges to one also in this case.

Note, finally, that the present discussion of diffraction in a more general context than Bragg’s law enables one to understand not just the position of the peaks but also their intensities. The particle positions in Fig. 8 ▸(*d*) were chosen to be identical to the 2D hexagonal arrangement of the pores in the experimental SAXS pattern in Fig. 4 ▸(*a*). Compared with the structure factor in Fig. 9 ▸(*c*), the measured second and third diffraction peaks seem to be almost extinct. This has nothing to do with a lack of structural periodicity, but merely results from the multiplication of the structure factor by the form factor of the pores, in line with equation (14). In the case of porous silica in Fig. 4 ▸(*a*), the form factor of the pores has a minimum at a value of *q* that is coincidentally very close to the peak position in the structure factor. When all is properly accounted for, the relative intensities of the peaks in Fig. 4 ▸(*a*) can be used to infer the pore size of the material (Gommes *et al.*, 2016 ▸).

## 5. Conclusion

Although the physics of X-ray or neutron interaction with matter can be complicated to understand in detail, many geometric aspects of it are well captured by the classical Huygens–Fresnel construction of Fig. 2 ▸. This makes the concepts of small-angle scattering easily understandable in a qualitative yet rigorous way with very little mathematics. We hope the present paper can contribute to making the technique more accessible and to promoting its use in the broad community of non-specialized scientists interested in nano­structured materials in general.

The principles of wave–matter interaction exposed here are more general than small-angle scattering as they apply to many other elastic scattering methods common in physical chemistry laboratories. The case of X-ray diffraction has been discussed explicitly, but other examples include molecular weight determination by static light scattering, the characterization of colloids by dynamic light scattering *etc*. The concepts of the paper will enable teachers to discuss these many techniques in a unified way, which will help students understand them in more depth and apply them more creatively.

## Supplementary Material

Animation to complement Fig. 7. DOI: 10.1107/S1600576721010293/gj5274sup1.mp4

## Figures and Tables

**Figure 10:**
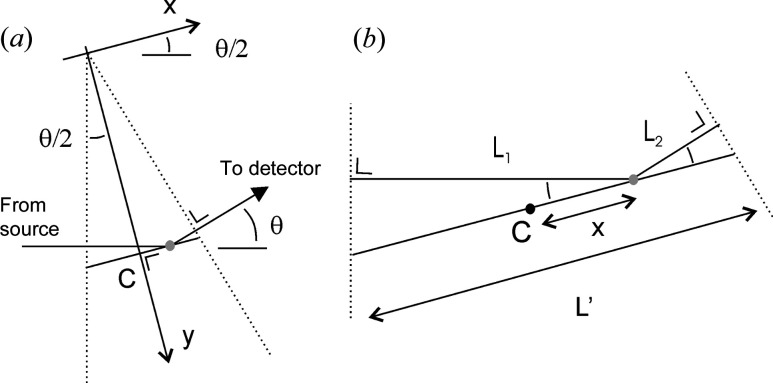
Calculation of the phase of a secondary wave when it reaches the detector (same configuration as in Fig. 2 ▸) as a function of the position of the scattering centre. The scattering centre is shown as a grey dot and its position is specified through axes (*x*, *y*) oriented as in panel (*a*). Point C is the intersection of the plane orthogonal to the *y* axis and passing though a point (*x*, *y*). (*b*) A magnified view of panel (*a*), in which the distances *L*′, *L*_1_ and *L*_2_ are defined.

**Table 1:** Examples of scattering exponents α connected to typical structures The values are not unique, but they provide hints at the underlying structure when analysing small-angle scattering data.

Scattering exponent α	Underlying structure
1	Randomly oriented elongated objects (needles, rods *etc.*)
2	Randomly oriented flat objects (platelets, discs *etc.*)
2	Ideal polymer coil, *i.e.* modelled as a random walk
∼1.7	Self-avoiding polymer coil
1 ≤ α ≤ 3	Volume fractals (aggregates, house of cards *etc.*) with fractal dimension *D* = α
3 < α < 4	Surface fractals, with fractal dimension *D*_s_ = 6 − α
4	Porod’s law: any structure with sharp interfaces between contrasted domains
4 < α	Smooth/progressive transitions between contrasted domains

## Appendix A The phase map ϕ_θ_(*x*, *y*, *z*)

We address here a geometric question. Considering Fig. 2 ▸, for a given position of the source and detector (strictly, of a given pixel in an actual detector), what is the length of a wave’s trajectory from the source to the detector, which passes through a given point (*x*, *y*, *z*)? To indicate that the answer depends on the detector position, we call this *L*_θ_(*x*, *y*, *z*). Because the phase of a wave increases by a quantity 2π each time it travels a distance equal to the wavelength λ, the phase is obtained as 2π*L*_θ_(*x*, *y*, *z*)/λ. This can also be expressed as measuring the distance between the source and detector via a scatterer, in units of λ/(2π).

To answer the question, we choose space coordinates with axes (*x*, *y*) as shown in Fig. 10 ▸(*a*), *i.e.* making an angle θ/2 with the incoming beam, and *z* orthogonal to both *x* and *y*. The source and detector are both assumed to be infinitely far away so that all incoming waves travel parallel to each other, as do all outgoing waves. This defines a wedge with opening angle θ, the left-hand side of which is at a distance *L*_S_ from the source, while the right-hand side is at a distance *L*_D_ to the detector. The grey dot in Fig. 10 ▸ is the position of a point (*x*, *y*, *z*) for which we endeavour to calculate *L*_θ_(*x*, *y*, *z*). With the self-explanatory notations of Fig. 10 ▸(*b*), the total distance travelled by a wave from the source to the detector, via the electron, is *L* = *L*_S_ + *L*_1_ + *L*_2_ + *L*_D_.

We use the notation *L*′ for the total opening of the wedge at distance *y* from the origin, as shown in Fig. 10 ▸(*b*). Using trigonometry, one finds *L*′/2 = *y*tan(θ/2). From Fig. 10 ▸(*b*), it is also apparent that *L*_1_ = (*L*′/2 + *x*)cos(θ/2) and *L*_2_ = (*L*′/2 − *x*)cos(θ/2). Putting all this together leads to

The important thing about this equation is that the coordinate *x* cancels out. This means that all points on the line making an angle of θ/2 with respect to the incoming beam would lead to secondary waves reaching the detector with the same phase. This result remains true in three dimensions, if the lines are replaced by planes orthogonal to *y*.

In terms of the phase ϕ_θ_ = 2π*L*_θ_/λ, this leads to the following expression:

which is the result that we were looking for, represented in Fig. 5 ▸. Equation (3) results from observing that the first term in equation (16) is the irrelevant constant ϕ_0_, which depends only on the arbitrary position of the axes. The factor in front of *y* in the second term of equation (16) is the scattering wavevector *q*, defined in equation (1).

## Appendix B Incoherent scattering as a random walk in amplitude space

We elaborate here on the relation between incoherent scattering and random walks in a complex amplitude plane. If the position of the scatterers is random like in Fig. 8 ▸(*a*), the real and imaginary parts of the amplitude *A*_r_ = Re{*A*} and *A*_i_ = Im{*A*} are also random numbers. From equation (5), the real part is

where each phase ϕ is uniformly and independently distributed over [0, 2π). Each term in the sum has an average equal to 0 and a variance equal to *a* ^2^/2. If *N* is sufficiently large for the central-limit theorem to apply, the real part of the amplitude is therefore Gaussian distributed with variance *Na* ^2^/2. The probability density is

and the imaginary part *A*_i_ is identically distributed. As the intensity is  , the corresponding distribution is

with d*A*_r_d*A*_i_ = πd*I*. From this distribution the average intensity is 〈*I*〉 = *Na* ^2^, as in equation (11), and the percentiles can easily be calculated as reported in Fig. 8 ▸. Equation (19) implicitly assumes that *A*_r_ and *A*_i_ are statistically independent, which is justified in the limit of large *N* (Merzbacher *et al.*, 1977 ▸).

When expressed in terms of the amplitude |*A*| = *I* ^1/2^, the distribution in equation (19) is known as Rayleigh’s probability density function, and it was first proposed in the context of acoustics (Rayleigh, 1880 ▸; Merzbacher *et al.*, 1977 ▸). Much later, Lord Rayleigh suggested it as a solution to a statistical problem posed by Pearson (1905 ▸) to model the travelling of mosquitoes, thereby bringing it into the field of random walks. As recalled by Nahin (2009 ▸), Pearson commented, ‘I ought to have known it, but my reading of late years has drifted into other channels, and one does not expect to find the first stage in a biometric problem in a memoir on sound.’ It is a nice twist that this equation appears again in a wave propagation setting, not about acoustics but about X-ray and neutron scattering.
